# Comparative analysis of RUSLE and SWPT for sub-watershed conservation prioritization in the Ayu watershed, Abay basin, Ethiopia

**DOI:** 10.1016/j.heliyon.2024.e35132

**Published:** 2024-07-24

**Authors:** Baye Terefe, Tadele Melese, Fekadu Temesgen, Abebe Anagaw, Amene Afework, Girmaw Mitikie

**Affiliations:** a Department of Geography and Environmental Studies, Injibara University, Injibara, Ethiopia; b Space Science and Geospatial Institute, Adis Ababa University, Addis Ababa, Ethiopia; c Department of Natural Resource Management, Bahir Dar University, Bahir Dar, Ethiopia; d Department of Geography and Environmental Studies, Bahir Dar University, Bahir Dar, Ethiopia

**Keywords:** Ayu watershed, GIS, Remote sensing, Soil loss, RUSLE, Sub-watershed prioritization

## Abstract

Ethiopia is currently facing a major environmental problem caused by soil erosion. In order to tackle this problem, it is essential to implement a comprehensive watershed management approach and give priority to conservation efforts depending on the level of severity. Therefore, the objective of this research is to evaluate the mean annual soil erosion and rank the sub-watersheds for conservations in the Ayu watershed, utilizing the Revised Universal Soil Loss Equation (RUSLE) model and the Sub-Watershed Prioritization Tool (SWPT). RUSLE was utilized to predict the annual average soil erosion rate, while SWPT was applied to conduct Weighted Sum Analysis (WSA) for ranking sub-watersheds. Support Vector Machine (SVM) was employed for classifying land use and land cover. The Relative importance of morphometric and topo-hydrologic features in the SWPT was analyzed using a Random Forest model. The Bland-Altman plot and Wilcoxon Signed Rank Test were employed to assess the agreement in prioritizing watersheds between RUSLE results and the SWPT. Furthermore, field observations were conducted to validate the land use classification by collecting ground data. In addition, the study was enhanced with local viewpoints by conducting focus group discussions with agricultural experts and farmers to obtain qualitative insights and validation of resuts. The findings showed that soil loss varied from 0 to 110 t/ha/yr, with an average of 8.95 t/ha/yr, resulting in a total loss of 384365.3 tons annually. The comparison of RUSLE and SWPT showed a moderate positive relationship (r = 0.59). The results of the Bland-Altman plot indicate a consistent agreement between the two methods. However, there is inconsistency among the five sub watersheds. This study enhances the knowledge of soil erosion patterns and offers useful guidance for watershed conservation techniques. It can be also used as a beneficial framework for managing watersheds, with possible uses outside of the Ayu watershed.

## 1 Introduction

Soil is recognized as a non-renewable and highly vulnerable natural resource [[1], [2], [3]]. It undergoes a natural process known as soil erosion. This process involves the detachment and transport of soil particles by various agents, leading to the degradation of topsoil and, in severe cases, subsoil through water, tillage, or wind [[4], [5], [6], [7]]. This multifaceted global land degradation process is among the most challenging and continuous environmental problems [4] and adversely impacts ecosystem services and functions [8,9]. [5,8,9]Soil erosion would have also an impact on the fertility of agricultural land and the quality of water [10].

At the global level, water-induced soil erosion is the predominant contributor to soil deterioration [[11], [12], [13]]. Given soil's limited and scarce availability, effective soil resource management is crucial to meet current demands and ensure sustainability. However, widespread soil degradation has occurred in many parts of the world, with soil erosion emerging as a primary cause of land degradation, posing threats to the natural environment, agriculture, and the overall economy [[14], [15], [16], [17]]. Nationally, soil loss costs 23 % of the national annual GDP of Ethiopia [18].

The gradual erosion process, driven by water or wind, results in soil detachment, transportation, and deposition. Different forms of water erosion, including splash erosion, sheet erosion, rill erosion, gully erosion, and stream bank erosion, are commonly recognized in different parts of the earth's surface [19]. Runoff-induced soil erosion is particularly problematic worldwide, posing a severe threat to agriculture and the natural environment due to unsustainable land use practices.

In developing countries, the incapacity of farming populations to replace lost soils and nutrients intensifies the impact of soil erosion [[20], [21], [22], [23]]. Africa, for instance, experiences over two-thirds of farmland degradation due to soil erosion [24]. Soil erosion and nutrient depletion threaten food security and the sustainability of agricultural production in sub-Saharan Africa [14]. In the Ethiopian highlands, extending agriculture to marginal lands has heightened pressure on land, loss of soil nutrients, water resources [25], vegetation leading to watershed management issues [[26], [27], [28], [29]]. The annual soil erosion on the Ethiopian highlands is estimated at 1.5 billion tons [ 30].

Efforts to address these challenges began in the 1980s, with Ethiopia initiating watershed-based planning [31]. However, these initiatives faced challenges such as insufficient community participation, unmanageable planning units, and a lack of in-depth watershed studies. Understanding watershed characteristics, the degradation level and effective watershed management becomes crucial for maintaining river well-being and productive capacity [[32], [33], [34]].

The Ayu watershed, a significant contributor to the Great Blue Nile River, has been experiencing soil fertility degradation due to water erosion, visible through gully formation and sheet erosion. Overgrazing, deforestation, and steep slopes further contribute to the erosion issues in the study area.

Assessing soil erosion is a vital requirement for planning and conserving soil and water resources [16,[35], [36], [37], [38]]. In watershed management programs, where addressing the entire area simultaneously is impractical, prioritizing areas based on erosion risk becomes essential [22,39,40]. For the estimation of soil erosion and watershed prioritization, the integration of remote sensing, geographic information systems (GIS), and other field data collection techniques facilitates a multidisciplinary perspective in studying soil erosion. The use of remote sensing and GIS-compatible models, such as RUSLE, has been widely adopted to estimate soil erosion rates and map erosion risk areas in various studies [16,17]. While some studies have used the RUSLE model to prioritize watersheds based on the annual average rate of soil loss [13,16,17,22,41]. Morphometric analysis methods alone is also utilized for sub-watershed prioritizations [42].

A study conducted by Ref. [43]have employed both a morphometric analysis method in combination with RUSLE model for prioritization purposes. The other study tried to compare RUSLE based watershed prioritization and the other morphometric analysis method [44], however still there is insufficient literatures related with the comparison between RUSLE and SWPT approaches considering the Ayu watershed and the nearby watersheds.

Therefore, this study aims to fill this gap by scientifically estimating soil loss, prioritizing sub-watersheds using both RUSLE model and a multicriteria approach that considers morphometric and topo hydrological parameters, and proposing proper watershed management techniques for the Ayu watershed. By doing so, this study will contribute to a broader understanding of soil erosion at various geographical scales and provide alternative conservation planning options to address the frequent soil erosion issues in the Study watershed.

## 2 Materials and methods

### 2.1 Study area description

**Fig. 1:**
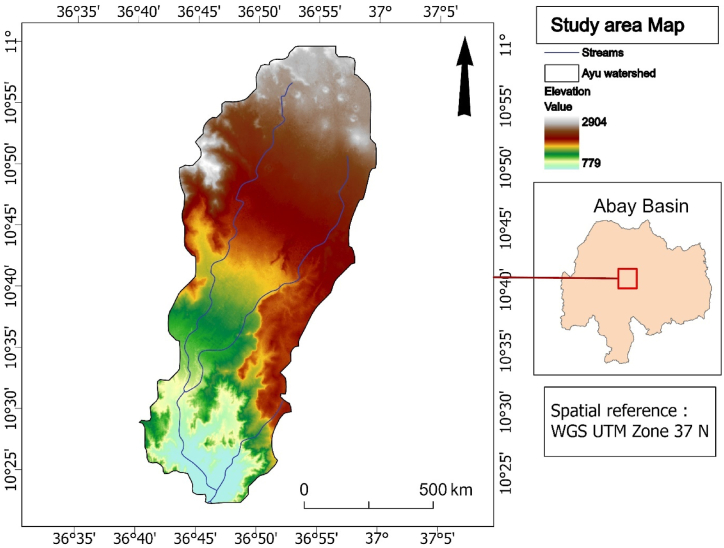
Location map of Ayu watershed (Source: Authors, 2023).

The study was conducted in the Ayu watershed, located in the upper part of Abay Basin, Northwestern Ethiopia. The coordinates of the study area lie between 10^0^^25^' N to 11^0^ 0̍' N latitude and 36^0^40̍' E to 37^0^0̍' E longitude, as illustrated in Fig. 1. The study area has a spatial coverage of 1065 km^2^ and the watershed is located around 440 km Northwest of Addis Ababa and forms the parts of the Abay River Drainage Basin. The study area has an elevation range from 779 to 2904 m above mean sea level (Fig. 1), with a topography that consists primarily of mountains, cliffs, and undulating terrain. The predominant soil types are Acrisols, Cambisols, fluvisols, leptosols, luvisols, nitosols, and vertisols [45].

The study area is divided into three climate zones. Kola (tropical), Woina Dega (sub-humid), and Dega (humid). The rainfall pattern is defined by a maximum rainfall pattern with maxima in July and August. It receives annual rainfall of 1778 mm and an average annual temperature of 18.82 °C, with mean monthly minimum and maximum temperatures of 10.12 °C and 27.98 °C, respectively (Ethiopian Meteorological Agency, 2022).

The study area's principal land uses and land covers are cultivated land, grassland, shrubs, forest land, and settlement area (Fig. 6). Mainly, the upper part of the watershed area is dominated by acacia ‘*decurence’* tree plantation [46].

### 2.2 Data sources and methods of data collection

The data used for this study were collected from primary and secondary sources. The primary data were ground truth points collected using Hand held GPS to validate land use land cover classification. Whereas the secondary data includes rainfall data, land use, digital elevation model (DEM), and digital soil data. The rain fall data for stations with in study watershed were obtained from the National Meteorological Agency of Ethiopia. The digital soil map was obtained from the Geospatial Institute of Ethiopia. Meanwhile, the acquisition date of the Sentinel 2A satellite images, which have a spatial resolution of 10*10 m, was from January 15, 2022, to February 20, 2022. These images were downloaded from the official website https://scihub.copernicus.eu/using the Google Earth Engine API. The median function in Google Earth Engine was utilized to load the most suitable image for further analysis. The SRTM DEM data with a spatial resolution of 20 m by 20m were obtained from the USGS website https://earthexplorer.usgs.gov/. The Sentinel 2A image was used for land use classification. The data was then processed via the cloud computing platform Google Earth Engine. 50 ground truth points were collected from each land use of the watershed. These ground truth data were utilized to generate the image classification accuracy assessment.

### 2.3 RUSLE model parameters

RUSLE model is the most extensively used model for estimating the rates of inter-rill and rill erosion [16]. It is used to compute annual soil loss combining the five parameters in GIS and remote sensing platforms [13,40]. The five parameters are presented here as follows.

#### 2.3.1 Rainfall erosivity (R factor)

According to Ref. [47], the product of storm kinetic energy (KE) and maximum 30-min intensity (EI30) determines the erosivity of rain. In ungauged watershed, when there is no enough data on rainfall intensity or kinetic energy to calculate rainfall erosivity R factor can be calculated using the average annual rainfall data of nearby weather stations [8]. It is recommended to use long term rain fall data for erosivity factor, For instance a study conducted at Gununo watershed, southern Ethiopia used Average annual rain fall of 32 years [48]. Therefore, in this study erosivity factor was calculated based on equation (1), considering 26 years of meteorological rainfall data (1996–2022) collected from 8 stations. The Inverse Distance Weighted (IDW) interpolation technique has been used to compute the rainfall data for the whole study area, as it is easy to define and easy to understand the results [40]. The rainfall erosivity factor has been computed for the study area using mean annual rainfall data from a linear equation adapted by Ref. [15] to Ethiopian circumstances, as given in Equation (1) below.

(1) R = −8.12 + (0.562 × P)

Where, P is the mean annual rainfall (mm) and R is the rainfall erosivity factor.

#### 2.3.2 Soil erodibility (K factor)

It is the natural resistance of soil to rainwater movement and soil particle detachment by rainwater. The physical and biological characteristics of the soil determine its erodibility, which ranges from 0 to 1, where the value 0 indicates soil has least susceptibility to erosion while 1 indicates soil has highly susceptible to soil erosion by water [5]. The absence of reliable data on soil properties significantly hinders larger geographic-scale soil erosion models. Based on soil type data in Table (1), K-factor values for use in Ethiopia were suggested to alleviate the need for exact laboratory analysis-based data on soil characteristics like texture and structure. This is due to the belief that the visible physical characteristics of the soil represents its attributes. Thus, in this study, the k value was determined using the type of soil in the watershed considered (Table 1).

**Table 1:** Soil type and K values of Ayu watershed.

Soil type	K factor	References
Acrisols	0.35	[5]
Cambisols	0.25	[7]
Fluvisols	0.15	[5]
Leptosols	0.21	[8]
Luvisols	0.26	[8]
Nitosols	0.25	[5]
Vertisols	0.14	[5]

#### 2.3.3 Slope length and steepness (LS factor)

The rise in L and S causes an increase in the LS factor and soil erosion, for which numerous methodologies of estimating the LS factor have been developed [49]. Several academics used different ways to compute the LS factor [22,50,51]. Commonly, these methods are used to calculate LS factor considering flow direction, flow accumulation, slope, flow length, and slope steepness [49,52]. In this study, a formula used by Refs. [22,53] was considered to calculate the LS factor(equation (2)).

(2) LS = ((FA*cell size)/ 22.13)^0.4^ * ((sin (slope in degree)) * 0.01745) / 0.0896) ^1.3^ * 1.4

Where LS denotes slope length and steepness, FA denotes flow accumulation, and Cell size (the spatial resolution of the DEM) denotes cell size.

#### 2.3.4 Cover management (C factor)

The C-factor denotes the influence of land cover and land management methods on soil erosion. It considers vegetation cover, crop type, agricultural residue management, and conservation practices, among other things [29,54]. It ranges between 1 and 0, where 1 indicates a lack of cover and a near-zero value indicates a very strong cover [55]. Most studies used Landsat data captured during the winter(dry season in Ethiopia), but [13] tried to use Landsat data captured during the summer when the crop cover is the highest in Ethiopia. Studies conducted in Ethiopia used different types of satellite image resolution to get high-resolution, for instance, spot 6 image was used by Ref. [56]. Among the 42 papers reviewed by Ref. [57] 37 of the articles used Land use land cover, and 5 articles used NDVI (Normalized Difference Vegetation Index) to compute the c factor values.

This study used land cover data classified using the Support vector machine classification method (SVM) based on sentinel 2A satellite data with a spatial resolution of 10m by 10m. Support vector machine provides better accuracy when training samples are small in number [58]. According to Afework et al. [46]; methodological review, the SVM image classification method is highly accurate and quite effective than the nearest neighbor, discriminant analysis, and maximum likelihood algorithms. Then, the C factor value was computed based on the information provided in Table 2.

**Table 2:** C- Factor value based on land cover.

Land Use/Land cover type	C-factor value	References
Farm Land	0.15	[16,20,51]
Forest	0.001	
Shrub	0.014	
Gras Land	0.010	
Settlement	0.09	
Water bodies	0	

#### 2.3.5 P-factor (support practice)

It considers the impact of erosion control strategies or conservation measures undertaken in a specific geographic area and considers conservation techniques like contour plowing, terracing, bunds, and other soil conservation strategies [4,59,60]. In the other way it can be also calculated based on the soil loss ratio from managed and cultivated fields, including up and down the slope [61]. The p-values range from 0 to 1, with 0 indicating well-managed fields and 1 indicating uncontrolled fields [62,63]. The P-factor value varies according to the adoption and effectiveness of conservation practices in various areas [15]. Different studies calculated p-factor value-based land use classes and slope categories [20,51]. This study calculated P factor value based on the agricultural land and other land use classes specified in Table 3.

**Table 3:** P factor Value.

Land Use	Slope class	P factor value	References
Agricultural land	0–5	0.1	[13,22]
	5–10	0.12	
	10–20	0.14	
	20–30	0.19	
	30–50	0.25	
	50–100	0.33	
Other land uses	All	1	

### 2.4 Estimation of annual average soil loss using RUSEL model

RUSLE is considered an empirical model [9]. It estimates soil loss based on observed relationships between soil erosion and various easily measurable factors without explicitly incorporating the underlying physical processes of erosion [32]. RUSLE uses a mathematical equation that considers slope length, slope steepness, rainfall erosivity, soil erodibility, cover management practices, and erosion control practices [15]. These factors are determined based on field and empirical data collected from various locations. While RUSLE incorporates some physical aspects, such as slope characteristics and land management practices, its primary approach is empirical. It calculates soil loss based on observed correlations between erosion and the input parameters. This empirical nature allows RUSLE to provide relatively simple and practical estimates of soil erosion that can be practical to different locations and conditions [9,32]. In Ethiopia's context, the RUSLE model utilizes specific parameters relevant to the country's soil and climatic conditions [12].

**Fig. 2:**
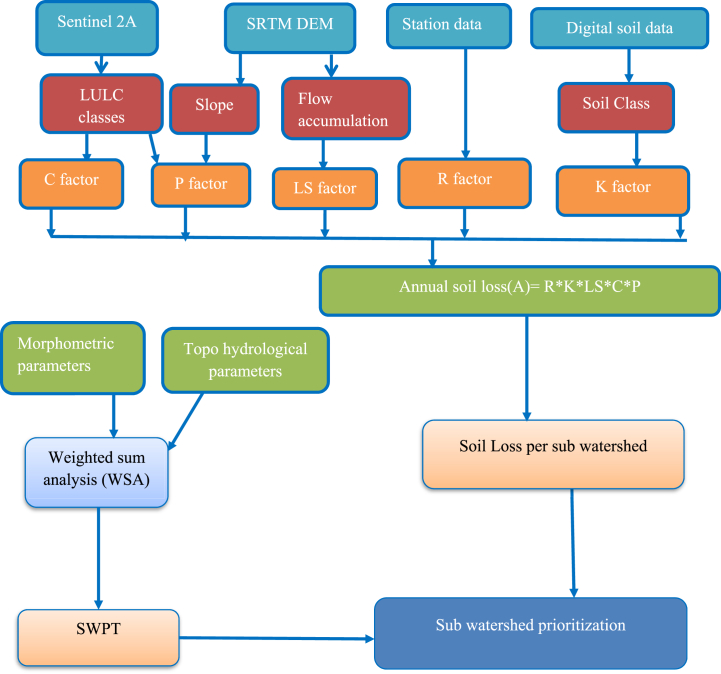
Methodological frame work.

The estimation of average annual soil loss within the Ayu watershed was conducted by integrating the computed results of the five crucial parameters in the RUSLE model (Fig. 2). After computing a raster layer, the annual average sheet and rill soil erosion is calculated by multiplying the following five parameters (equation (3)) using the raster calculator under the spatial analysis tool package of ArcGIS pro 3.0 environment. The classification of mean annual soil loss into severity classes presented the following distribution in percentage terms based on range classified by Bekele et al., [22].

(3) A = R *K* LS* C* P

where A stands for annual soil loss (t ha−^1^ year−^1^);

R stands for rainfall erosivity factor (MJmmha−^1^h−^1^ year−^1^);

K stands for soil erodibility factor (t ha−^1^MJ−^1^mm−^1^);

L stands for slope length, and S is slope steepness factor(dimensionless);

C stands for cover and management factor(dimensionless), and P is support and conservation practice factor(dimensionless) [15].

### 2.5 Sub-watershed prioritization methods

Due to financial, human resource, and time restrictions, implementing successful Soil and water conservation (SWC) measures across the whole watershed is difficult. As a result, separating the entire area into priority categories based on the severity of erosion in the watersheds is useful [64,65]. The identification of erosion-risky regions is required when deciding where to focus conservation strategy and effort. The annual soil loss statistics from the RUSLE model are commonly used to prioritize and implement conservation practices at watershed level [22,48]. In this study, RUSLE outputs were classified per sub watersheds using Zonal Statistics as table tool in Arc GIS pro 3.0 software, the result was used for watershed prioritization purpose.

Another technique used in this study to prioritize sub watersheds was Weighted Sum Analysis (WSA) technique through SWPT (Fig. 2). It is a simple tool used simplify the data scarcity problem and are utilized for ungauged data integrated with GIS software. It takes into account morphometric and topo-hydrological parameters [66]. The SWPT technique uses statistical correlation to evaluate the relative relevance of each parameter, and it assigns weight to each parameter based on its due importance equation (4).

(4) $$\text{Prioritization}=\sum_{i=0}^{n}\;Wi*Xi$$

where Wi is the weight of each morphometric parameter determined using the SWPT technique, and Xi is the morphometric parameter value. The aforementioned approach is capable of recognizing the efficiency of components while taking individual effects into account.

The Ayu watershed, comprising 80 sub-watersheds, underwent classification using an Arc Hydro tool extension in the ArcGIS Pro 3.0 software (Fig. 9). To simplify the WSA watershed prioritization approach a high-level python programing based SWPT (sub watershed prioritization tool) was used to integrate 12 parameters which determine watershed prioritization processes (Table 4). The Random Forest model was used to analyze the relative importance of various features of morphometric and topo-hydrologic properties of sub-watersheds. The model assessed several properties and ranked them based on their contribution to predicting a target variable [67,68].

**Table 4:** Computation of morphologic and topo-hydrological parameters.

Parameters	Definition/formula
Stream frequency (Fs)	Fs = Nu/A, where Nu is total number of stream segments of order ‘u’ and A is area enclosed within the boundary of watershed divide (Basin area)
Compactness constant (Cc)	Cc = 0.2821P/A^0.5^, where P is length of watershed divide which surrounds the basin (Basin perimeter)
Constant of channel maintenance (C)	C = 1/D, where D is drainage density
Bifurcation ratio (Rb)	Rb = Nu/Nu_+1_ where N_u+1_ is number of segments of the next higher order
Drainage density (D)	D = Lu/A where Lu is total stream length of order ‘u’
Elongation ratio (Re)	Re =√(4*(A/pi)/Lb)/ where L_b_ is distance between outlet and farthest point on the basin boundary (Basin length)
Circularity ratio (Rc)	Rc = 4 * Pi * A/P2, where P is length of watershed divide which surrounds the basin (Basin perimeter)
Form factor (Rf)	Rf = A/Lb. 2 where Lb. is distance between outlet and farthest point on the basin boundary (Basin length)
Drainage texture ratio (Rt)	Rt = Nu/P
Topographic wetness index (TWI)	TWI = ln (As/tanβ) where ‘As’ is the local upslope area draining through a certain point per unit contour length and tanβ is the local slope
Stream power index (SPI)	As = tanβ
Stream transport index (STI)	STI= (m+1) * As/22.13^m^ *sinβ/0.0896^n^ where β is the local slope gradient in degrees, m is the contributing area exponent, and n is the slope exponent

The SWPT result was then evaluated based on the results of the compound parameter value (CPV). Prioritization is given to the sub-watershed with the lowest CPV value, and all other sub-watersheds are ranked accordingly [66].

### 2.6 Methods for the comparison of watershed prioritization based on RUSLE and SWPT

This study used correlation analysis to compare sub watershed priorization based on RUSLE outputs and SWPT. The Bland-Altman plot, a widely recognized method for assessing agreement between two quantitative measurements was used to visually examine the concordance between the RUSLE and SWPT methods for sub-watershed prioritization. It was originally introduced by Bland and Altman in 1986, this plot facilitates the exploration of consistency and potential bias between two methods by graphing the difference between their measurements against their mean [69]. Each data point on our Bland-Altman plot represents a sub-watershed, with the x-axis portraying the mean of the RUSLE and SWPT ranks, and the y-axis indicating the disparity between the RUSLE and SWPT ranks. We included a dashed line to represent the mean difference between the ranks, providing a reference point for any systematic bias. Then, Wilcoxon signed-rank test was used to compare the correlation between the RUSLE-based watershed rank and the SWPT watershed rank. This non-parametric test compared paired observations by assessing the difference in ranks.

### 2.7 Model validation

The RUSLE model, which was used to assess annual soil erosion in the Ayu watershed, was supposed to be verified and calibrated using field measurements, erosion plots, and comparisons with observed data. It is known that the model operates effectively at plot and small catchment scales, demonstrating reasonable agreement between projected and actual soil loss [70]. This study validated RUSLE model based on comparisons with published literatures conducted in Ethiopia [12,71]. Additionally, the outputs were discussed with agricultural experts having field experiences within the watershed [22,72].

## 3 Results and discussions

This section shows the findings of our in-depth investigation into annual soil erosion quantification in the Ayu watershed, situated within the Blue Nile Basin in Ethiopia. The analysis centered on the estimation of soil loss and comparisons of RUSLE output based and SWPT watershed prioritization.

### 3.1 RUSLE factor analysis

The in-depth analysis of the RUSLE model parameters—rainfall erosivity (R factor), soil erodibility (K factor), cover factor (C factor), topographic factor (LS factor), and practice factor (P factor)—has provided detail understanding of their impacts on soil erosion. The analysis results are presented in the following subsections.

#### 3.1.1 Rainfall erosivity (R factor) analysis

**Fig. 3:**
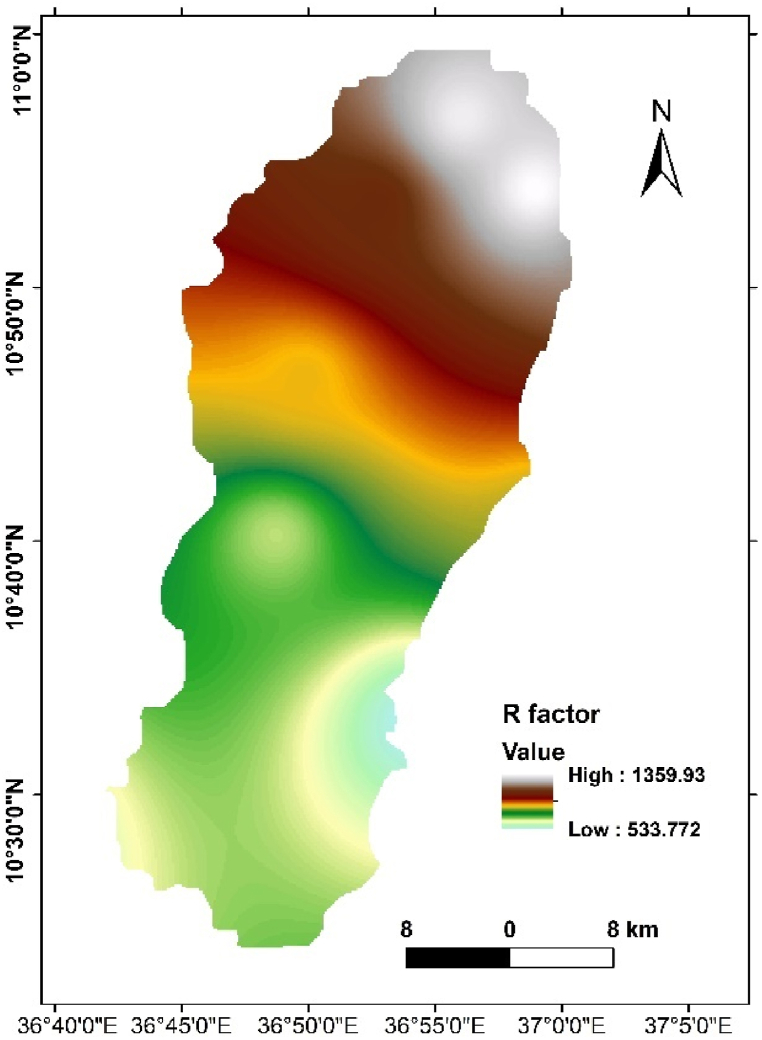
Rainfall erosivity (R factor) map of Ayu watershed.

The Rainfall Erosivity (R Factor) analysis results indicated a notable range of R factors, varying between 533.77 and 1359.93 MJ mm ha^−1^ year^−1^ (Fig. 3). This variability underscores the diverse erosivity potential within the watershed. Considering the mean annual rainfall data, our findings emphasize the climatic conditions contributing to the erosive potential within the Ayu watershed. This comprehensive Rainfall Erosivity analysis not only contributes to the understanding of soil erosion dynamics but also lays the groundwork for informed and context-specific land management practices within the study area.

#### 3.1.2 Soil erodibility (K factor) analysis

**Fig. 4:**
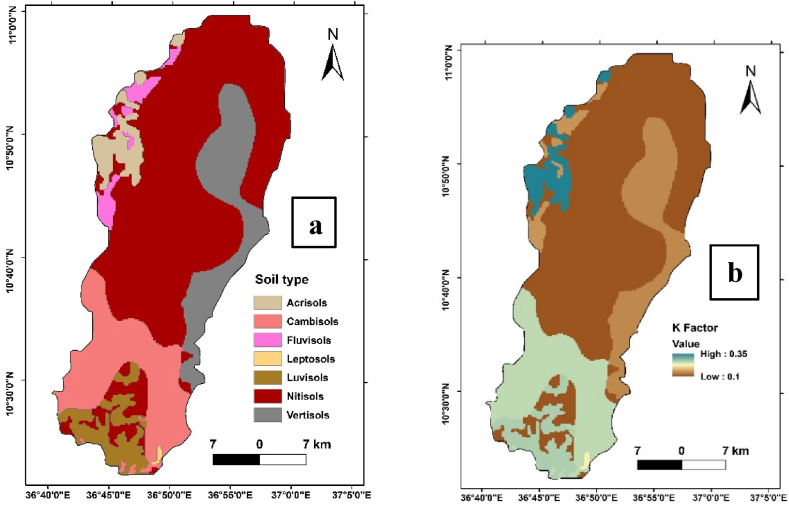
Soil type map of Ayu watershed (a), Soil erodibility (K factor) map of Ayu watershed(b).

The K factor, representing the susceptibility of soil to erosion, ranged from 0.1 to 0.35 across the diverse soil types present in the Ayu watershed (Fig. 4 a&b). This range signifies the variability in erodibility potential, with certain soil types exhibiting a higher tendency for erosion compared to others. The distinct characteristics of Acrisols, Cambisols, Fluvisols, Leptsols, Luvisols, Nitosols, and Vertisols played a pivotal role in determining the corresponding K factor values.

The soil erodibility analysis provides crucial insights into the vulnerability of different soil types to erosive forces within the Ayu watershed. Understanding the K factor variation allows for the development of targeted soil conservation strategies suited to the specific soil properties encountered across the landscape. These findings contribute to a more detailed approach to sustainable land management practices, emphasizing the importance of soil type in mitigating soil erosion risks within the study area.

#### 3.1.3 Topographic factor (LS factor) analysis

**Fig. 5:**
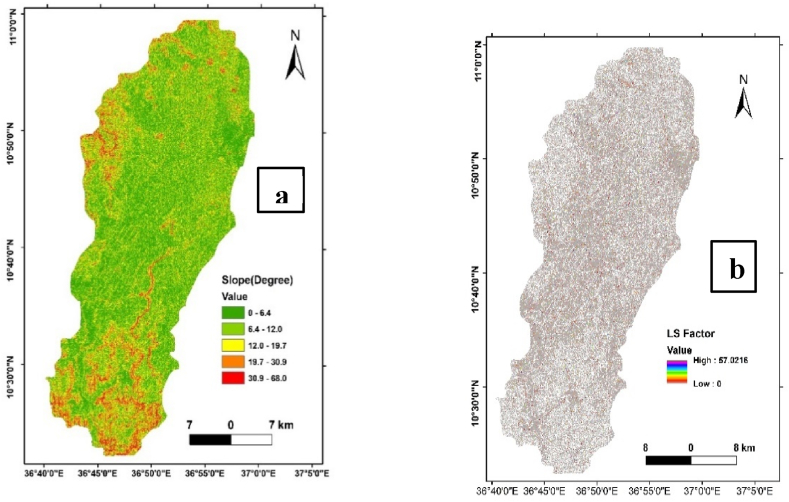
Slope map of Ayu watershed (a), Topographic (Ls) factor map of Ayu watershed(b).

The slope values were determined in degrees to align with the requirements of the applied formula. The resulting slope dataset provided a comprehensive representation of the terrain, ranging from 0 to 68°, reflecting the diverse topography within the Ayu watershed (Fig. 5a).

Subsequently, the LS factor, a crucial element in the Revised Universal Soil Loss Equation (RUSLE) model, was computed using a raster calculator based on Equation (2). This calculation involved the integration of slope, considering both its magnitude and length, to assess the impact of topography on soil erosion potential. The LS factor values, derived from this analysis, exhibited a range from 0 to 31.7021 across the study area (Fig. 5b). The LS factor values offer valuable insights into the influence of topography on soil erosion susceptibility within the Ayu watershed. Higher LS factor values indicate areas with steeper slopes and longer flow paths, suggesting elevated erosion potential. Conversely, lower LS factor values signify areas with less pronounced topographic influence, indicating a reduced risk of soil erosion. This detailed topographic factor analysis contributes essential information for land management decisions, aiding in the identification of priority areas for erosion control measures within the study area**.**

#### 3.1.4 Cover factor (C factor) analysis

**Fig. 6:**
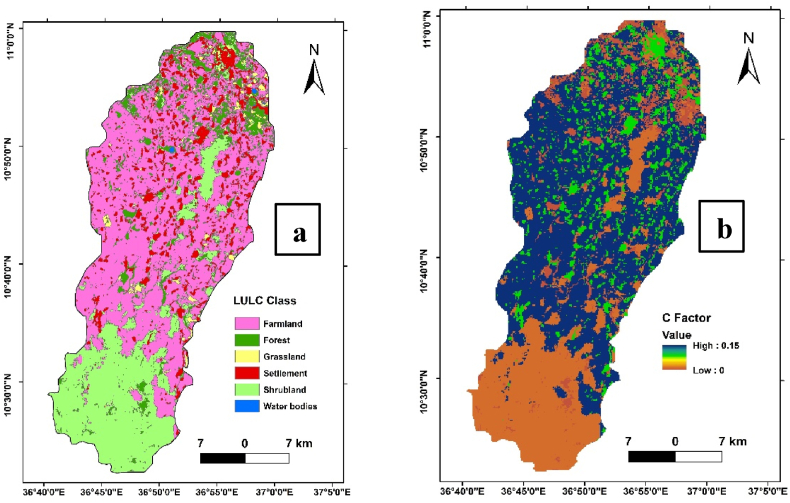
Land use-land cover map of the Ayu watershed(a), cover factor result of Ayu watershed(b).

The computation of the C factor revealed values ranging from 0 to 1.5 across the Ayu watershed (Fig. 6a and b). These values denote the varying degrees of soil cover and management influence on soil erosion potential within different land cover types. Lower C factor values suggest effective soil and cover management practices that mitigate erosion, while higher values indicate areas with increased vulnerability to soil loss.

Understanding the C factor distribution across different land cover types is crucial for implementing targeted land management strategies. It facilitates the identification of areas requiring conservation efforts and highlights the effectiveness of existing land use practices in mitigating soil erosion risks. The results of the C factor analysis contribute valuable insights for sustainable land management and erosion control initiatives within the Ayu watershed.

#### 3.1.5 Conservation practice factor (P factor) analysis

**Fig. 7:**
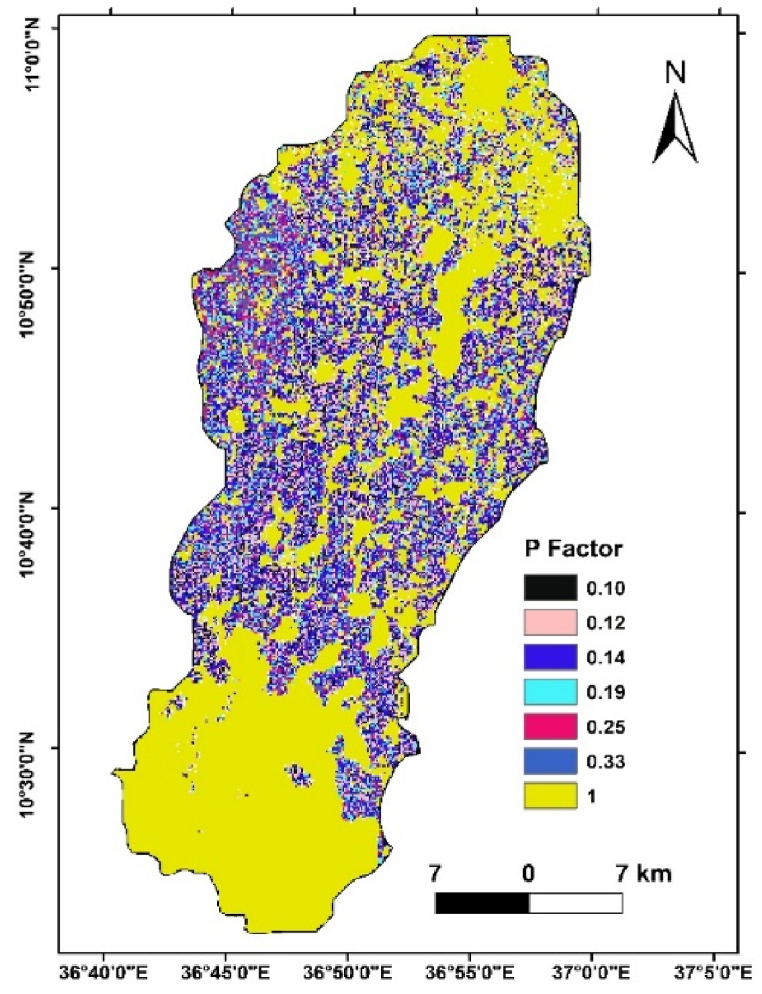
Conservation practice factor (P factor) map of Ayu watershed.

The calculated P factor values exhibited a range from 0.10 to 1, as depicted in Fig. 7. This range signifies the effectiveness of conservation practices across the Ayu watershed, with lower values, such as 0.10, indicating areas where conservation practices are more impactful in mitigating soil erosion. Conversely, higher P factor values may suggest areas with limited or less effective conservation practices.

While the ideal approach involves utilizing direct data on conservation practices, the reliance on land cover data offers a pragmatic solution in the absence of specific conservation data. The P factor analysis provides valuable insights into the effectiveness of current land management practices within the watershed. Understanding the spatial distribution of P factor values aids in identifying areas where targeted conservation efforts can be prioritized to enhance soil erosion control measures and promote sustainable land use practices.

### 3.2 Potential average annual soil loss estimation using RUSLE model

The in-depth analysis of the RUSLE model parameters—rainfall erosivity (R factor), soil erodibility (K factor), cover factor (C factor), topographic factor (LS factor), and practice factor (P factor)—has provided detail understanding of their impacts on soil erosion. The combined effects underscore the importance of considering the holistic influence of these parameters in the integrated watershed management and conservation.

The results revealed an annual average soil loss ranging from 0 to 110 t/ha/yr. with average value of 8.95 t/ha/yr. The total annual soil loss was 384,365.3 tons from the Ayu watershed. The range signifies the spatial variability of soil erosion susceptibility across the Ayu watershed. This result is consistent with studies reported that the average annual soil loss was from 5 to 20 t/ha/yr [51,55,71,73]. These studies mentioned that the forest contributes for the low risk of soil erosion by water The other study reported a higher annual soil loss rate greater than 50 t/ha/yr [20,50,74]. These studies stated that the severity of soil erosion was facilitated by the extensive deforestation and topographic factors in their study area.

**Fig. 8:**
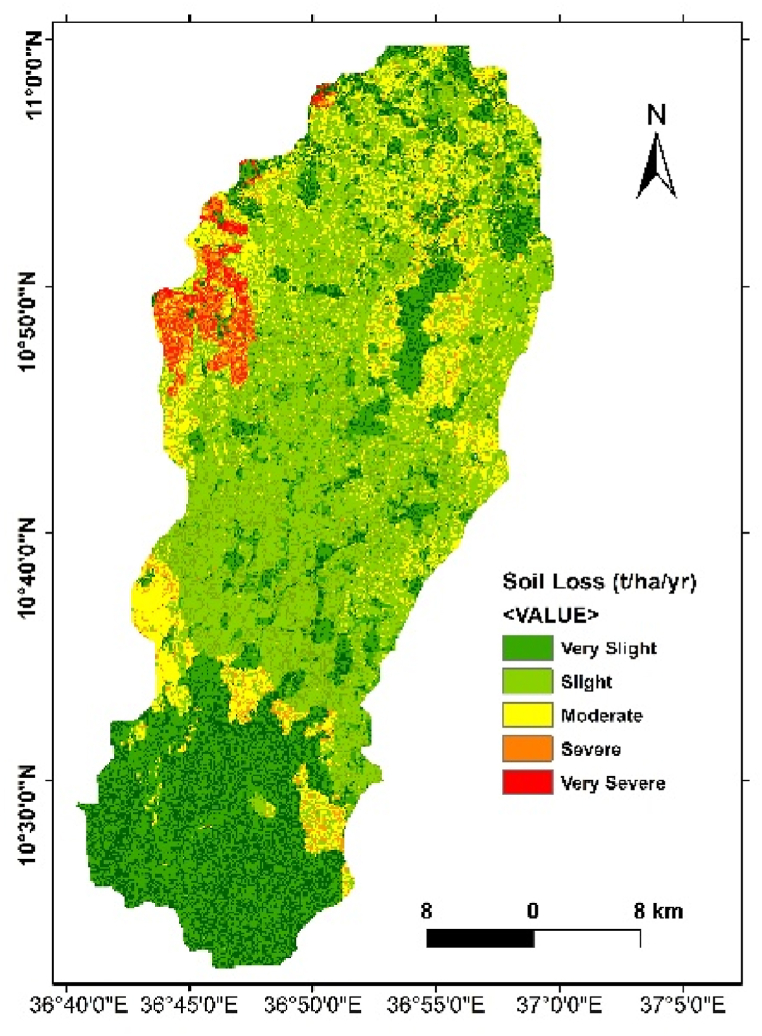
Map of severity classes based on average annual Soil loss rate of Ayu watershed.

To facilitate interpretation, the mean annual soil loss rates were classified into five severity classes: very low severity, low severity, moderate severity, and high severity (Fig. 8). The distribution of soil loss exhibited substantial spatial variation in different directions within the Ayu watershed.

**Fig. 9:**
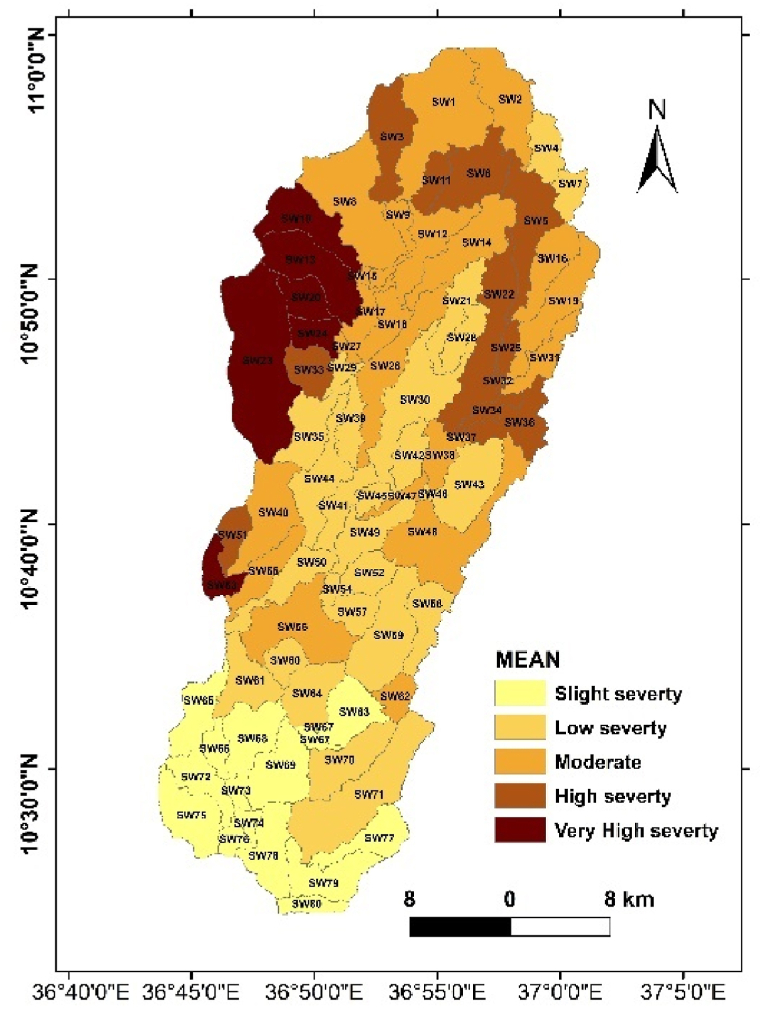
Map of Ayu watershed average annual soil loss rate classified per the sub watersheds.

The classification of mean annual soil loss into severity classes presented the following distribution in percentage terms based on range classified by Bekele et al. [22],: Slight severity (33.4 %), low severity (37.8 %), moderate severity (20.4 %), and high severity (6.1 %) and very high severity (2.3 %). These percentages elucidate the distribution of different soil erosion intensities across the watershed, offering critical insights for targeted conservation and land management efforts. These findings not only enhance our understanding of the soil erosion dynamics within the Ayu watershed but also provide a foundation for implementing effective measures to mitigate soil loss and promote sustainable land use practices.

### 3.3 Average annual soil loss rate at the sub watershed level

The results indicate significant variations in soil loss rates across the sub-watersheds. The result indicated that the mean annual soil loss with in the Ayu watershed is extremely diversified, where the north western parts of the watershed is highly vulnerable to soil erosion and there is low soil loss rate recorded in the lower watershed area (Fig. 9). These spatially varying distribution of soil loos rates across the study area emphasizes the necessity for localized and context specific soil conservation strategies [75,76]. The observed variations in the severity classes highlight areas with distinct erosion intensities, providing a foundation for targeted conservation and management efforts. This spatial awareness is necessary for developing effective measures to mitigate soil loss and promoting sustainable land use practices which fits with the specific soil erosion levels of each sub watersheds with in the study area [77,78].

The mean annual soil loss rate played a pivotal role in watershed prioritization, with the study utilizing this information to prioritize sub-watersheds based on erosion risk [38,39,61]. The results of the study were also compared with those obtained using the Sub-Watershed Prioritization Tool (SWPT), revealing the synergistic importance of combining soil loss rates with prioritization tools [79,80]. The validity of the findings was affirmed through focus group discussions with agricultural experts and farmers within the watershed, providing qualitative insights for the identification of severity of soil loss rate at sub watershed level.

### 3.4 Prioritization of sub-watersheds based on morphometric and topo-hydrological parameters

The SWPT analysis report yielded a diverse array of correlations among the morphometric and topo-hydrological properties within the sub-watersheds. Stream Frequency (Fs) exhibited significant positive correlations with Stream Density (D) and Form Factor (Rt), while notably displaying a strong negative correlation with the Stream Transport Index (STI). Form Factor (Rf) demonstrated a robust positive correlation with Elongation Ratio (Re) and displayed positive correlations with Rc and SPI. In contrast, Rf exhibited negative correlations with Compactness Constant (Cc) and Topographic Wetness Index (TWI). Elongation Ratio (Re) displayed a very strong positive correlation with both Rf and Rc, while concurrently manifesting a negative correlation with Cc. Compactness Constant (Cc) presented negative correlations with Rf, Re, Rc, and SPI, accompanied by a positive correlation with TWI. Drainage Density (D) displayed positive correlations with Fs and Rt, juxtaposed with negative correlations with Constant of Channel Maintenance (C) and Stream Transport Index (STI). Drainage Texture Ratio (Rt) demonstrated negative correlations with C and STI, while simultaneously exhibiting positive correlations with Fs and D. Topographic Wetness Index (TWI) was positively correlated with Cc and inversely correlated with Fs, D, Rt, STI, and SPI. Stream Power Index (SPI) displayed positive correlations with Rc and STI, and concurrently revealed negative correlations with Cc and TWI (Table 5). These morphometric and topo hydrologic parameters can be easily extracted from DEM data using GIS software's. They have tremendous influences on the erosional process of a watershed. For instance according to Sharma et al. [79], as Rb increases, flood damage is more likely to occur. A watershed with a greater Rf value achieves a peak runoff rate/flow in a small period, whereas a watershed with a lower Rf value results in a flow for longer periods with a flatter peak. RC has an impact on stream characteristics, a circular river basin outperforms an elongated basin in terms of surface runoff discharge. A lower Re value implies severe erosion and sediment load susceptibility, while a higher Elongation ratio value suggests strong infiltration capacity with minimal runoff. Higher SPI values indicate greater erosive power and sediment transport potential of the flowing water. The parameters are not only the natural features,they are affected by vegetative cover, rainfall, lithology, infiltration capacity, and the relief characteristics of the basin [65,79,80].

**Table 5:** Correlation matrix of morphometric and topo-hydrologic properties for the sub-watersheds.

	Fs	Rb	Rf	Re	Rc	D	Rt	Cc	C	TWI	SPI	STI
Fs	1	−0.02	0.26	0.25	0.03	0.95	0.97	−0.07	−0.55	0.04	−0.3	−0.72
Rb	−0.02	1	−0.3	−0.31	−0.13	−0.04	−0.05	0.13	0.12	−0.03	0.09	0.2
Rf	0.26	−0.3	1	0.99	0.49	0.28	0.34	−0.47	−0.25	−0.17	0.07	−0.33
Re	0.25	−0.31	0.99	1	0.5	0.27	0.33	−0.48	−0.24	−0.19	0.09	−0.31
Rc	0.03	−0.13	0.49	0.5	1	0.03	0.11	−0.95	−0.02	−0.63	0.61	0.2
D	0.95	−0.04	0.28	0.27	0.03	1	0.98	−0.06	−0.75	0.1	−0.36	−0.82
Rt	0.97	−0.05	0.34	0.33	0.11	0.98	1	−0.14	−0.68	0.02	−0.29	−0.78
Cc	−0.07	0.13	−0.47	−0.48	−0.95	−0.06	−0.14	1	0.01	0.62	−0.59	−0.19
C	−0.55	0.12	−0.25	−0.24	−0.02	−0.75	−0.68	0.01	1	−0.2	0.4	0.8
TWI	0.04	−0.03	−0.17	−0.19	−0.63	0.1	0.02	0.62	−0.2	1	−0.95	−0.5
SPI	−0.3	0.09	0.07	0.09	0.61	−0.36	−0.29	−0.59	0.4	−0.95	1	0.72
STI	−0.72	0.2	−0.33	−0.31	0.2	−0.82	−0.78	−0.19	0.8	−0.5	0.72	1

#### 3.4.1 Feature importance ranking of morphometric and topo-hydrological parameters

**Fig. 10:**
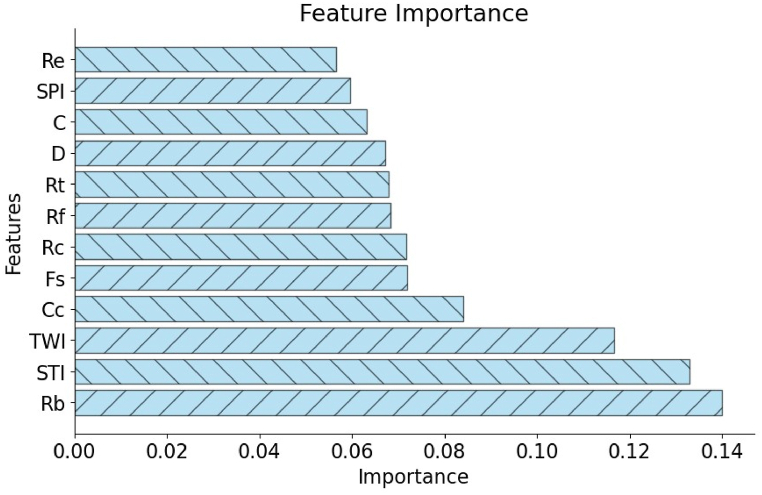
The importance of morphometric and topo-hydrologic features in predicting watershed outcomes.

In watershed prioritization for conservation, understanding the importance of different parameters is crucial. Feature importance ranking quantitatively assesses the contribution of individual parameters in prioritization processes. This subsection explores the significance of feature importance ranking in morphometric and topo-hydrological analysis for watershed conservation (Fig. 10).

The feature ranking revealed that the Rb is the most critical feature with an importance score of 0.1399, followed by the STI with a score of 0.1329, and the TWI at 0.1167. These top three properties significantly impact watershed characteristics, suggesting they play a crucial role in hydrological and geomorphological processes [79]. Further down the ranking, the Cc scored 0.0841, and Fs at 0.0719, highlighting their moderate importance. The Rc and Rf both had similar importance scores of 0.0717 and 0.0683, respectively, indicating their role in watershed shape and runoff characteristics. Other properties such as the Rt, D, and C had importance scores ranging from 0.0679 to 0.0631, emphasizing their relevance but to a lesser extent. The SPI and Re were also included in the analysis, with lower importance scores of 0.0596 and 0.0566, respectively. A bar plot (Fig. 10) visualizing these feature importance underscores the dominance of Rb, STI, and TWI, providing clear guidance on which properties should be prioritized in watershed management and further studies. This ranking can aid in understanding the critical factors influencing watershed health and behavior, ultimately supporting better decision-making in environmental management practices.

The compound parameter value (CPV) was used to prioritize sub-watersheds, taking into consideration the subtle interactions between morphometric and topo-hydrologic features (Table 6). The observed correlations provide vital insights into the interconnections of various metrics, allowing for a more complete knowledge of the Ayu watershed's characteristics.

**Table 6:** Prioritization and final ranking of sub-watersheds based on SWPT result.

Watershed	CPV	Rank	Watershed	CPV	Rank	Watershed	CPV	Rank
SW1	−27.81947943	5	SW30	−22.04330092	13	SW59	−27.97022745	4
SW2	−22.38551568	11	SW31	−14.90812616	41	SW60	−13.46856743	49
SW3	−15.65889376	35	SW32	−4.936296824	78	SW61	−19.22879278	20
SW4	−15.49595909	36	SW33	−15.01382867	40	SW62	−12.36070205	61
SW5	−15.08809549	39	SW34	−10.62833478	64	SW63	−18.65127289	23
SW6	−18.47761588	24	SW35	−18.38971811	25	SW64	−19.5389876	19
SW7	−11.5471367	63	SW36	−18.8786497	21	SW65	−17.34062589	27
SW8	−25.12623695	6	SW37	−3.63266005	80	SW66	−9.924064816	66
SW9	−8.08796566	75	SW38	−14.4855833	42	SW67	−8.667419164	74
SW10	−22.82365088	10	SW39	−14.22971458	43	SW68	−20.28058887	17
SW11	−13.7635486	45	SW40	−12.85565077	55	SW69	−21.84842689	14
SW12	−18.30820792	26	SW41	−15.91485974	33	SW70	−19.9819367	18
SW13	−23.01163129	8	SW42	−13.08752222	50	SW71	−30.35832402	3
SW14	−16.95684557	30	SW43	−23.84486773	7	SW72	−17.26255166	28
SW15	−5.357516131	77	SW44	−14.19700615	44	SW73	−10.04887372	65
SW16	−15.12664409	38	SW45	−9.563024542	69	SW74	−9.808545252	67
SW17	−13.65531739	47	SW46	−4.130512553	79	SW75	−21.44435539	15
SW18	−12.77519468	56	SW47	−9.523533746	70	SW76	−9.783269272	68
SW19	−17.13380491	29	SW48	−30.49883345	2	SW77	−18.70563818	22
SW20	−13.05112024	52	SW49	−12.6167239	58	SW78	−22.88630705	9
SW21	−12.36663986	60	SW50	−16.54279093	31	SW79	−20.74855383	16
SW22	−15.3122164	37	SW51	−12.32471947	62	SW80	−12.49480852	59
SW23	−31.4429952	1	SW52	−13.66389197	46			
SW24	−13.00391486	54	SW53	−13.05945774	51			
SW25	−9.472757395	71	SW54	−9.074345944	72			
SW26	−13.64886384	48	SW55	−15.85817715	34			
SW27	−5.502784177	76	SW56	−22.33381625	12			
SW28	−13.0289676	53	SW57	−12.62274014	57			
SW29	−8.684340488	73	SW58	−16.47139812	32			

* SW= Sub watershed, CPV (Compound parameter value), rank (prioritization rank), each sub watershed is shown in Fig. 9.

**Fig. 11:**
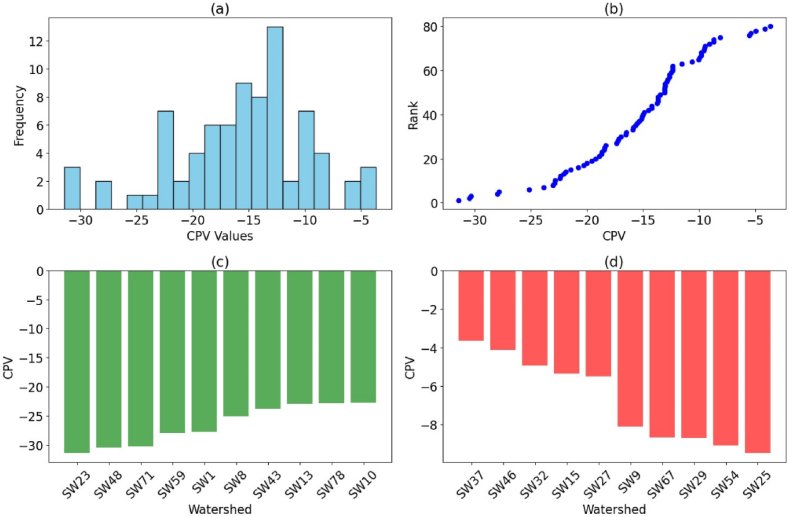
CPV value interpretation visual analysis of sub-watershed prioritization (a) histogram distribution of CPV across sub-watersheds (b) Scatter plot of CPV vs. rank showing the inverse relationship (c) bar plot of the top 10 ranked sub-watersheds with lowest CPV scores (d) bar plot of the bottom 10 ranked sub-watersheds with highest CPV scores.

The histogram of CPV across the sub-watersheds demonstrates a wide range of CPV scores (Fig. 11a). This variability indicates significant differences in the morphometric and topo-hydrological characteristics of the sub-watersheds. The distribution appears to be skewed, with a higher frequency of sub-watersheds having lower CPV values. This skewness suggests that most of the sub-watersheds have relatively lower CPV, which can be interpreted as having better morphometric and hydrological conditions. The scatter plot of CPV versus Rank provides insight into the prioritization process (Fig. 11b). As expected, there is an inverse relationship between CPV and Rank; sub-watersheds with lower CPV scores tend to have higher ranks, while those with higher CPV scores have lower ranks. This inverse relationship confirms the effectiveness of using CPV as a metric for prioritizing sub-watersheds based on their morphometric and topo-hydrological properties.

The bar plot highlighting the top 10 ranked sub-watersheds reveals the best-performing sub-watersheds based on their CPV scores (Fig. 11c). Sub-watershed SW23 ranks the highest with the lowest CPV score of −31.44, indicating it has the most favorable characteristics among all sub-watersheds. The other top-ranked sub-watersheds also show significantly low CPV scores, reinforcing their prioritization. These sub-watersheds should be considered as benchmarks or models for sustainable watershed management practices due to their superior morphometric and hydrological conditions. Conversely, the bar plot of the bottom 10 ranked sub-watersheds illustrates the sub-watersheds that perform the worst based on their CPV scores (Fig. 11d). Sub-watershed SW32 ranks the lowest, with a CPV score of −4.94, indicating it has the least favorable characteristics. The other sub-watersheds in this category also have relatively high CPV scores. These sub-watersheds may require more focused management efforts to improve their morphometric and hydrological conditions. Identifying these sub-watersheds can help prioritize resource allocation for rehabilitation and conservation measures [62,64,79].

The Compound Parameter Value (CPV) for prioritizing sub-watersheds, considering the interconnections of various features, further enhances our knowledge for effective watershed management planning. These approach of watershed prioritization is cost effective [66]. However, this approach has limitations which does not consider the human intervention on the watershed.

### **3.5** Comparison of sub-watershed prioritization based on RUSLE and SWPT

**Fig. 12:**
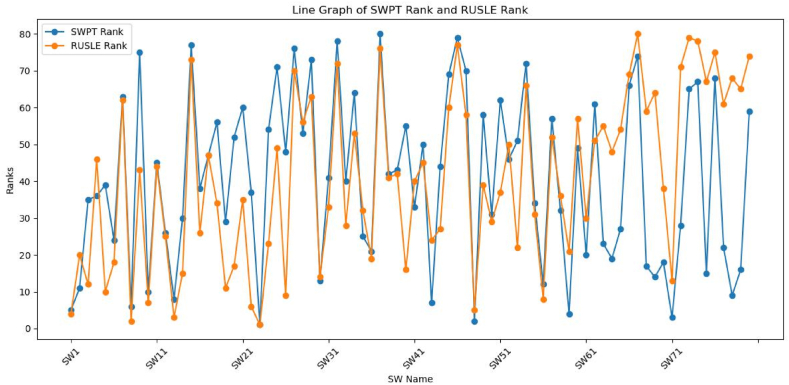
Correlation between RUSLE and SWPT prioritization result.

The results of the comparative analysis between RUSLE and SPWT revealed a moderate positive correlation (r = 0.59) between the sub-watershed prioritization based on soil loss rate and the WSA approach applied using sub watershed prioritization tool (Fig. 12). The first method, which considered land cover and land management practices, provided insights into the impact of human activities on soil erosion. It identified sub-watersheds with high soil loss rates due to factors such as deforestation, improper land use, and inadequate conservation practices. On the other hand, the second method, utilizing elevation data, highlighted sub-watersheds with vulnerability due to its morphometric and topo-hydrological characteristics**.** This method focused on the natural topographic characteristics that contribute to erosion vulnerability, intrinsically keeping the influence of human activities [64,79].

The comparison enhances the precision of identifying erosion risk areas, demonstrating the synergistic importance of combining these approaches for effective watershed management. Studies conducted by Refs. [22,41,81] prioritized watershed based on the annual soil loss rate. Where the other study prioritized combining soil loss rate estimated using RUSLE model and other multicriteria based models [23].

**Fig. 13:**
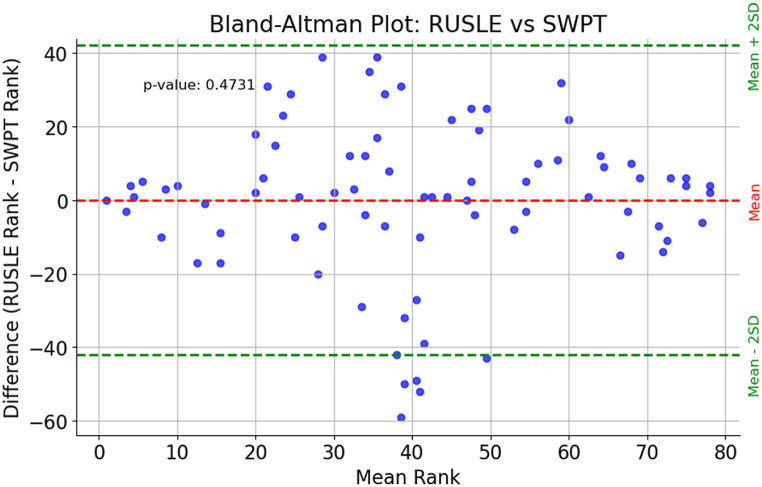
A Bland-Altman plot comparing the RUSLE and SWPT methods for sub-watershed prioritization.

The findings reveal a generally consistent alignment between the two methods, as evidenced by the clustering of data points around the mean difference line (Fig. 13). This observation echoes previous research emphasizing the robustness of both RUSLE and SWPT in evaluating watershed characteristics. The limits of agreement, defined as the mean difference ±2 times the standard deviation of the differences, offer a range within which most data points should ideally fall to demonstrate strong agreement between the methods [69].

The Wilcoxon Signed-Rank Test was conducted to compare the rankings obtained from the RUSLE method with those from the SWPT method. The test yielded a statistic of 1396.5 and a p-value of 0.4731. With a significance threshold of 0.05, the p-value exceeds this threshold, indicating no significant difference between the RUSLE and SWPT rankings. Therefore, we fail to reject the null hypothesis, suggesting that the median difference between paired ranks is zero. This implies that both methods produce comparable rankings for sub-watersheds, supporting the notion of their agreement in prioritization.

#### 3.5.1 Sub-watersheds with significant ranking discrepancies

There were some differences in the 80 sub watershed rankings done based on RUSLE and SWPT findings (Table 2). Considering these differences is crucial for effective management and allocation of resources when selecting watersheds for conservation efforts. There is debate about the key factors affecting watershed health, evident in the varied rankings of sub-watersheds based on different attributes or standards. Understanding the reasons behind these differences is crucial for ensuring that conservation efforts are targeted at the most critical areas and taking the necessary steps.

**Fig. 14:**
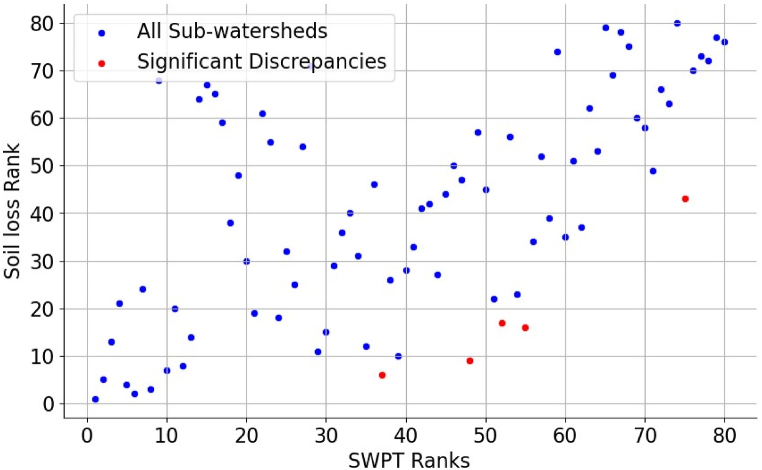
RUSLE and SWPT rankings and their discrepancies.

The above result in Fig. 14 shows a significant but not perfect agreement between the two methods in ranking sub-watersheds, indicating that areas with higher rankings in one method tend to have higher rankings in the other method as well. Nevertheless, even with this general consensus, there are significant differences in the rankings of specific sub-watersheds between RUSLE and SWPT. Sub-watersheds like SW40, SW26, SW20, SW9, and SW22 show notable variations in their rankings, with discrepancies ranging from 31 to 39 ranks. These differences point out where the two approaches differ in what they prioritize, possibly showing areas of doubt or fluctuation in the data or modeling assumptions. In this study the discripancies are happened in areas where there is lower accuracy of land use land cover classification which was used to copmute RUSLE parameters including C and P factors (Fig. 6, Fig. 7). This diccripsncy was checked through field observstion by cross validating the analysis results. To sum up, though there is generally some agreement between RUSLE and SWPT rankings, finding sub-watersheds with significant ranking differences emphasizes the need for additional evaluation and validation of watershed prioritization methods considering high resolution satellite images and advanced deep learning techniques, which allows to allevaite the limitations related with C and P factors of RUSLE model. More research on the factors causing these differences can offer valuable knowledge for enhancing the precision and dependability of prioritization methods in watershed management and conservation endeavors.

### 3.6 Validation of model outputs

The validation of any model result using ground data plays a critical role in research results report. However, the absence of measured soil loss and sedi-ment yield data in the study area necesitated other alternatives. Accordingly, in this study the outputs of RUSLE model was validated comparing with previous research conducted in similar agroecology. For instance, a research conducted at Zingin watershed reported 9.1 t/ha/yr. which is in line with the findings of this study [82]. Other studies also reported the mean annual soil loss which ranges within 12–13 t/ha/yr [55,83]. Field observations were used to identify erosion-prone areas, with field trips showing the model's findings using printed maps and qualitative discussions with stockholders, similar approach was also used in a research conducted in the highlands of Ethiopia [22,41,72].

## 4 Conclusions

This research sought to evaluate soil erosion in the Ayu watershed, prioritize sub-watersheds for conservation, and offer practical inputs for sustainable soil and water conservation. The RUSLE model and SWPT were utilized for estimating annual soil loss and determining priority sub-watersheds for conservation initiatives. The research revealed that the annual average soil erosion rate in the Ayu watershed varied between 0 and 110 t/ha/yr., averaging at 8.95 t/ha/yr., leading to an annual soil loss of 384,365.3 tons. The SWPT and RUSLE model showed a moderate positive correlation to prioritize sub watersheds for conservation, but differed in ranking five sub-watersheds. The SVM model was applied for land use and land cover classification, and gathering stakeholder viewpoints involved conducting focus group discussions with agricultural experts and farmers.

The study is limited by only focusing on one watershed, possible limitations in the methods used to compute the cover and practices factors used in RUSLE model, and the subjective nature of stakeholder viewpoints. Future studies should take into account numerous watersheds for comparison, enhance the quality of computation of cover and practice factors through and advanced deep learning techniques, and utilize a more rigorous sampling method for gathering input from stakeholders. The results of the study have implications for how soil erosion and watershed conservation are prioritized and managed, offering important information for policymakers and land managers to create successful plans for controlling soil erosion and managing watersheds. Leaders can use the findings of the research to create specific plans, highlight key sub-watersheds for protection, and effectively use proven modeling methods. Finally, the research highlights the importance of combining watershed management and focusing on conservation efforts, offering a useful outline for managing watersheds outside of the Ayu watershed.

## Data availability statement

Not applicable.

## Additional information

No additional information is available for this paper**.**

## CRediT authorship contribution statement

**Baye Terefe:** Writing – review & editing, Writing – original draft, Visualization, Validation, Resources, Project administration, Methodology, Investigation, Funding acquisition, Formal analysis, Data curation, Conceptualization. **Tadele Melese:** Writing – review & editing, Methodology, Conceptualization. **Fekadu Temesgen:** Writing – review & editing, Methodology, Funding acquisition, Formal analysis, Conceptualization. **Abebe Anagaw:** Writing – review & editing, Resources, Methodology, Funding acquisition, Conceptualization. **Amene Afework:** Writing – review & editing, Methodology, Conceptualization. **Girmaw Mitikie:** Writing – review & editing, Funding acquisition, Conceptualization.

## Declaration of competing interest

The authors declare that they have no known competing financial interests or personal relationships that could have appeared to influence the work reported in this paper.

## Figures and Tables

**Table 1:** Morphometric and topo-hydrological parameters of the sub-watersheds in Ayu watershed

Name	Fs	Rb	Rf	Re	Rc	D	Rt	Cc	C	TWI	SPI	STI
**SW1**	1.65E-06	5.921569	0.28847	0.606054	0.174691	0.001685	0.00137	2.392008	593.4915	9.808942	5.223722	13.5408
**SW2**	3.27E-06	2.116667	0.275738	0.592529	0.158191	0.002145	0.001883	2.51366	466.1411	9.60599	5.209128	12.30924
**SW3**	6.94E-06	1.682527	0.244873	0.558382	0.188217	0.003184	0.004116	2.304454	314.0339	9.536718	5.214935	12.24076
**SW4**	6.11E-06	1.942708	0.529807	0.821335	0.24128	0.003176	0.002967	2.035343	314.895	9.639434	5.161304	11.99992
**SW5**	5.62E-06	1.968168	0.274038	0.5907	0.095253	0.003343	0.00252	3.239357	299.1173	9.760258	4.97302	11.55988
**SW6**	4.19E-06	2.180147	0.357159	0.674361	0.173905	0.002631	0.002386	2.397405	380.1393	9.679189	5.015338	11.61443
**SW7**	1.19E-05	2.199561	0.706956	0.948763	0.254566	0.004349	0.004788	1.981517	229.9522	9.64278	5.001906	10.86405
**SW8**	1.66E-06	1.854167	0.401613	0.715097	0.188061	0.00189	0.001366	2.305414	529.0393	9.662756	5.107976	12.50891
**SW9**	3.96E-05	1.994109	0.298255	0.616248	0.08961	0.006955	0.009727	3.339792	143.7909	9.765792	4.799771	9.970396
**SW10**	3.65E-06	2.432692	0.275481	0.592253	0.176236	0.002094	0.002073	2.381498	477.4978	9.20645	5.615894	12.86462
**SW11**	9.16E-06	2.041667	0.261775	0.577332	0.175466	0.003666	0.00318	2.386718	272.8026	9.643098	5.00375	11.42063
**SW12**	3.82E-06	1.875	0.266351	0.582356	0.112406	0.00269	0.001601	2.981963	371.7387	9.732528	4.960505	11.99401
**SW13**	2.97E-06	1.96875	0.39228	0.70674	0.21791	0.00207	0.002069	2.141705	483.0219	9.250344	5.632824	12.83354
**SW14**	4.07E-06	2.489583	0.450728	0.757563	0.127857	0.002899	0.002135	2.795994	344.9226	9.701332	4.986731	12.00269
**SW15**	8.71E-05	2.505952	0.72249	0.959129	0.237506	0.010637	0.014236	2.05145	94.00886	9.537057	4.956615	9.196655
**SW16**	4.64E-06	2.083333	0.225041	0.535294	0.183705	0.003324	0.002331	2.332588	300.8166	9.862512	4.864154	12.39528
**SW17**	9.85E-06	2.420455	0.192563	0.495163	0.108285	0.003735	0.002638	3.038182	267.7594	9.809671	4.884908	11.21384
**SW18**	1.01E-05	1.855392	0.311079	0.629356	0.172878	0.003987	0.004082	2.404518	250.8078	9.78849	4.88847	11.06019
**SW19**	4.36E-06	2.416667	0.293078	0.610876	0.104291	0.002888	0.001959	3.095819	346.3018	9.878882	4.802503	11.58855
**SW20**	6.80E-06	2.25	0.36162	0.678559	0.338412	0.003846	0.003657	1.7186	259.9891	9.063605	5.842828	13.10386
**SW21**	1.05E-05	1.64881	0.529057	0.820753	0.126879	0.004123	0.003994	2.806745	242.5335	9.737873	4.870625	10.78628
**SW22**	5.89E-06	3.180147	0.288499	0.606085	0.084033	0.00327	0.002771	3.448842	305.7675	9.849022	4.829572	11.37536
**SW23**	1.33E-06	2.145833	0.268703	0.584922	0.26029	0.001489	0.001559	1.959609	671.6972	9.249468	5.671309	13.72059
**SW24**	8.57E-06	2.041667	0.432653	0.742218	0.273349	0.003847	0.003887	1.912225	259.9372	9.465065	5.310732	11.50013
**SW25**	2.27E-05	1.844298	0.441132	0.749455	0.175032	0.005586	0.006313	2.389675	179.0308	9.606632	4.955572	10.56007
**SW26**	8.54E-06	2.509821	0.248175	0.562135	0.081867	0.003747	0.003671	3.494162	266.9113	9.904927	4.739466	10.97468
**SW27**	9.19E-05	2.305263	0.508552	0.804691	0.15925	0.010778	0.015936	2.505294	92.78242	9.84428	4.570413	8.816379
**SW28**	1.08E-05	2.404167	0.272991	0.58957	0.159287	0.0039	0.003852	2.504998	256.4415	9.816055	4.813086	11.13134
**SW29**	2.98E-05	1.747857	0.619177	0.88791	0.149282	0.006155	0.006832	2.587586	162.4643	9.821437	4.724675	9.898526
**SW30**	2.58E-06	2.2	0.188797	0.490297	0.079778	0.002206	0.00122	3.539608	453.2123	9.905175	4.778587	12.04201
**SW31**	7.96E-06	2.108553	0.145097	0.429824	0.10517	0.003407	0.002503	3.082849	293.5104	9.85019	4.84049	11.76852
**SW32**	0.000108	2.597619	0.238439	0.550998	0.159935	0.012967	0.013655	2.499921	77.12101	9.683505	4.88826	9.548374
**SW33**	6.21E-06	2.25	0.520416	0.814023	0.339856	0.003264	0.003551	1.714946	306.3804	9.597915	5.182444	11.78963
**SW34**	2.07E-05	2.432609	0.494173	0.793233	0.125719	0.004878	0.005074	2.819663	205.0093	9.678335	4.914963	10.52017
**SW35**	4.26E-06	2.270833	0.242702	0.555902	0.169929	0.002657	0.002093	2.425291	376.3592	9.747912	5.000334	11.83668
**SW36**	5.14E-06	2.255208	0.29842	0.616418	0.124236	0.00259	0.002197	2.836438	386.1196	9.837305	4.938142	11.88295
**SW37**	0.000236	2.122585	0.608115	0.879943	0.22914	0.018263	0.026151	2.088564	54.75433	9.524525	4.700248	8.2882
**SW38**	8.88E-06	2.077206	0.3381	0.656121	0.182448	0.003442	0.003426	2.340607	290.5659	9.675405	5.004684	11.18834
**SW39**	7.93E-06	2.421875	0.195479	0.498898	0.140944	0.003552	0.002635	2.663017	281.5171	9.916511	4.710489	11.26345
**SW40**	7.25E-06	2.19958	0.499759	0.797704	0.18883	0.003935	0.004597	2.300711	254.1476	9.748609	5.082993	11.91848
**SW41**	5.64E-06	2.208333	0.205646	0.511708	0.126289	0.003144	0.002035	2.81329	318.073	9.900933	4.750634	11.68005
**SW42**	1.16E-05	2.607895	0.306213	0.624415	0.210636	0.003844	0.005077	2.178368	260.1614	9.571737	5.170045	11.44592
**SW43**	2.96E-06	2.075758	0.514794	0.809615	0.243478	0.001982	0.002102	2.026134	504.4625	9.616581	5.10013	11.65249
**SW44**	7.64E-06	2.908333	0.290165	0.607832	0.104665	0.003549	0.003289	3.090282	281.7613	9.798292	4.850914	11.2277
**SW45**	1.97E-05	1.592647	0.433726	0.743138	0.227343	0.005497	0.005967	2.096801	181.9037	9.854595	4.676554	9.957915
**SW46**	0.000197	1.983836	0.512523	0.807827	0.215411	0.015382	0.023563	2.154091	65.01035	9.467745	4.650511	8.117694
**SW47**	2.77E-05	2.775362	0.294486	0.612342	0.097183	0.005649	0.005261	3.207035	177.0304	9.802827	4.727492	10.38235
**SW48**	1.73E-06	1.916667	0.472127	0.775337	0.093434	0.001543	0.00097	3.27073	647.993	9.903521	4.926294	12.03469
**SW49**	8.87E-06	1.912037	0.291112	0.608824	0.141523	0.004082	0.003832	2.657567	245.0047	9.684706	5.104607	11.92166
**SW50**	5.37E-06	2.541667	0.306337	0.624542	0.106021	0.002995	0.001996	3.070447	333.9025	9.86554	4.79247	11.34321
**SW51**	7.80E-06	2.125	0.245184	0.558737	0.257928	0.004144	0.003324	1.968561	241.3042	9.390393	5.419076	12.28544
**SW52**	7.69E-06	2.067708	0.466566	0.770758	0.316926	0.003625	0.004014	1.775901	275.8243	9.424448	5.372367	11.58891
**SW53**	8.12E-06	1.929487	0.323866	0.642161	0.207663	0.003882	0.003152	2.193906	257.5684	9.910411	4.756886	11.18906
**SW54**	2.27E-05	2.125	0.511934	0.807362	0.264036	0.005761	0.006662	1.945656	173.5704	9.455761	5.195331	10.58764
**SW55**	5.65E-06	2.391667	0.160027	0.451396	0.137125	0.003158	0.002207	2.699852	316.6155	9.937499	4.806546	11.85085
**SW56**	2.10E-06	2.208333	0.46265	0.767516	0.156146	0.002144	0.00138	2.530068	466.3632	9.743997	5.012178	12.31485
**SW57**	8.88E-06	2.25	0.610712	0.88182	0.328115	0.003939	0.004517	1.74536	253.894	9.360377	5.483127	11.83749
**SW58**	5.72E-06	2.054167	0.567734	0.850225	0.179841	0.002971	0.002669	2.357509	336.5809	9.951004	4.777397	11.20166
**SW59**	2.70E-06	2.55	0.266384	0.582393	0.229111	0.001681	0.002047	2.088695	594.9302	9.484232	5.350368	12.43715
**SW60**	9.50E-06	1.416667	0.698602	0.94314	0.315236	0.003666	0.004367	1.780655	272.7992	9.279299	5.471196	11.59932
**SW61**	4.15E-06	2.278846	0.349214	0.666818	0.175982	0.00252	0.002102	2.383219	396.8708	9.708544	5.088105	11.82784
**SW62**	1.25E-05	1.401754	0.828273	1.026948	0.267464	0.004022	0.005185	1.933147	248.6364	9.540315	5.231683	10.90304
**SW63**	4.12E-06	1.850962	0.535598	0.825812	0.29141	0.002578	0.002624	1.85202	387.9167	9.027947	5.892365	12.70988
**SW64**	4.01E-06	1.880952	0.55216	0.838482	0.332611	0.002445	0.002765	1.733523	408.9397	9.020699	5.817397	12.55763
**SW65**	4.67E-06	1.989583	0.621967	0.889908	0.207411	0.002798	0.002453	2.195242	357.427	9.477964	5.293389	12.01648
**SW66**	1.80E-05	1.944444	0.276844	0.593717	0.228873	0.005308	0.00625	2.089781	188.3782	9.572602	5.269979	11.06676
**SW67**	2.23E-05	1.904167	0.496627	0.795201	0.160618	0.006181	0.004722	2.494597	161.7921	9.275796	5.421741	10.90775
**SW68**	3.83E-06	1.979167	0.378883	0.694567	0.252805	0.002369	0.002404	1.988406	422.0963	9.285656	5.534776	12.564
**SW69**	3.29E-06	1.983333	0.378577	0.694286	0.293145	0.002184	0.002447	1.846532	457.8996	9.085645	5.811811	13.11281
**SW70**	3.81E-06	2.059524	0.413153	0.725299	0.228844	0.002406	0.002386	2.089912	415.6604	9.281727	5.577236	12.47668
**SW71**	1.65E-06	2.321429	0.212269	0.519882	0.175863	0.001549	0.001333	2.384023	645.7363	9.152034	5.723628	13.60096
**SW72**	5.25E-06	1.9375	0.32167	0.63998	0.191592	0.002844	0.002417	2.284068	351.5942	9.300675	5.508079	12.74849
**SW73**	1.99E-05	2.375	0.380011	0.6956	0.302243	0.005156	0.006231	1.818528	193.962	9.382346	5.332944	11.4433
**SW74**	2.50E-05	1.457143	0.381173	0.696663	0.241511	0.005354	0.006509	2.034368	186.7611	9.410754	5.222306	10.94926
**SW75**	3.64E-06	2.034615	0.43713	0.746048	0.285543	0.002221	0.002539	1.870949	450.1783	8.84276	5.992164	13.10168
**SW76**	1.82E-05	2.216667	0.543904	0.83219	0.313827	0.005268	0.006546	1.784649	189.8186	9.1184	5.714897	11.8062
**SW77**	5.28E-06	2.125	0.391768	0.706279	0.181618	0.00259	0.002503	2.345945	386.1629	8.733675	6.111261	13.2736
**SW78**	3.17E-06	2.041667	0.348208	0.665857	0.24469	0.002081	0.001974	2.021111	480.5038	8.888652	5.959466	13.41286
**SW79**	3.98E-06	2.005208	0.313583	0.631884	0.186077	0.002322	0.002158	2.317672	430.6313	8.794896	6.045693	13.56172
**SW80**	1.25E-05	5.444444	0.210705	0.517963	0.247716	0.003978	0.00447	2.008729	251.3807	8.606726	6.281063	13.3962

## References

[1] Schoonover J.E., Crim J.F. (2015). An introduction to soil concepts and the role of soils in watershed management. J. Contemp. Water Res. Educ..

[2] Clunes J. (2022). Soil fragility: a concept to ensure a sustainable use of soils. Ecol. Indicat..

[3] Mancho Alonso C. (2024). Organic amendments application effect on a contaminated soil with arsenic. Rev. Int. Contam. Ambient..

[4] Gashaw T., Tulu T., Argaw M. (2017). Erosion risk assessment for prioritization of conservation measures in Geleda watershed , Blue Nile basin , Ethiopia. Environ. Syst. Res..

[5] Endalamaw N.T., Moges M.A., Kebede Y.S., Alehegn B.M., Sinshaw B.G. (2021). Potential soil loss estimation for conservation planning , upper Blue Nile. Environ. Challenges.

[6] Getnet T., Mulu A. (2021). Assessment of soil erosion rate and hotspot areas using RUSLE and multi-criteria evaluation technique at Jedeb watershed , Upper Blue Nile , Amhara Region , Ethiopia. Environ. Challenges.

[7] Sinshaw B.G. (2021). Watershed-based soil erosion and sediment yield modeling in the Rib watershed of the Upper Blue Nile Basin , Ethiopia. Energy Nexus.

[8] Abebaw L., Nagy A., Kendie H. (2022). Soil loss estimation and severity mapping using the RUSLE model and GIS in Megech watershed , Ethiopia.

[9] Luvai A., Obiero J., Omuto C. (2022). Soil loss assessment using the revised universal soil loss equation (RUSLE) model. Appl. Environ. Soil Sci..

[10] Singh G., Panda R.K. (2017). Grid-cell based assessment of soil erosion potential for identi fi cation of critical erosion prone areas using USLE , GIS and remote sensing : a case study in the Kapgari watershed , India. Int. Soil Water Conserv. Res..

[11] Yesuph A.Y., Dagnew A.B. (2019). Soil erosion mapping and severity analysis based on RUSLE model and local perception in the Beshillo Catchment of the Blue Nile Basin. Environ. Syst. Res..

[12] Almaw A., Tsunekawa A., Haregeweyn N., Tsubo M. (2021). Agroecology-based soil erosion assessment for better conservation planning in Ethiopian river basins. Environ. Res..

[13] Negese A., Fekadu E., Getnet H. (2021).

[14] Girmay G., Moges A., Muluneh A. (2020). Estimation of soil loss rate using the USLE model for Agewmariayam Watershed , northern. Agric. Food Secur..

[15] Hurni H. (1985).

[16] Abathun M., Yonas M., Hagos G., Malede D.A. (2022). Assessment of soil loss rate using GIS – RUSLE interface in Tashat Watershed , Northwestern Ethiopia. J. Sediment. Environ..

[17] Balabathina V.N., Raju R.P., Mulualem W., Tadele G. (2020). Estimation of soil loss using remote sensing and GIS - based universal soil loss equation in northern catchment of Lake Tana Sub - basin , Upper Blue Nile Basin , Northwest Ethiopia. Environ. Syst. Res..

[18] Ejegu M.A., Yegizaw E.S. (2021). “Modeling soil erosion susceptibility and LULC dynamics for land degradation management using geoinformation technology in Debre Tabor district. Northwestern highlands of Ethiopia,”.

[19] Morgan R.P. (2005).

[20] andDessalegn B.B.M., Moisa O.G., Badasa Mitiku, Niguse Dejene Indale (2022). “Soil loss estimation and prioritization using geographic information systems and the RUSLE model : a case study of the Anger River sub-basin. Western Ethiopia,”.

[21] Girma R., Gebre E. (2020). Spatial modeling of erosion hotspots using GIS - RUSLE interface in Omo - gibe river basin , Southern Ethiopia : implication for soil and water conservation planning. Environ. Syst. Res..

[22] Bekele D.A., Gella G.W., Ejigu M.A. (2022). Erosion risk assessment: a contribution for conservation priority area identification in the sub-basin of Lake Tana, north-western Ethiopia. Int. Soil Water Conserv. Res..

[23] Mhiret D.A., Dagnew D.C., Assefa T.T. (September, 2018). Erosion hotspot identification in the sub-humid Ethiopian highlands Ecohydrology & Hydrobiology Erosion hotspot identification in the sub-humid Ethiopian highlands. Integr. Med. Res..

[24] Hurni H. (1988). “Degradation and conservation of the resources in the Ethiopian highlands author (s): hans hurni source : mountain research and development. African Mountains and Published by : International Mountain Society Stable.

[25] Tamene L., Adimassu Z., Aynekulu E., Yaekob T. (2017). Estimating landscape susceptibility to soil erosion using a GIS-based approach in Northern Ethiopia. Int. Soil Water Conserv. Res..

[26] Masha M., Yirgu T., Debele M. (2021).

[27] Yiferu Y., Girma Taddese T., Mebrate (2018). “Influence of soil erosion and conservation practices on soil physical properties in Ginaberet.

[28] Demeke G.G., Andualem T.G., Tabor D., Engineering W.R., Tabor D. (2019). Modeling of soil loss and identification of erosion hot spot areas using RUSLE integrated with GIS for appropriate conservation practices in muga watershed, highlands of Ethiopia.

[29] Lemma E., Getahun Y.S., Getachew T. (2022). Prioritization of erosion hotspot microwatersheds for conservation planning using GIS and remote sensing techniques in antsokia-gemiza district of north shewa. Appl. Environ. Soil Sci..

[30] Haregeweyn N., Yohannes F. (2003). Testing and evaluation of the agricultural non-point source pollution model (AGNPS) on Augucho catchment , western Hararghe , Ethiopia.

[31] Alemu W.G., Melesse A.M. (2020). Impacts of longterm conservation measures on ecosystem services in Northwest Ethiopia. Int. Soil Water Conserv. Res..

[32] Regasa M.S., Nones M., Adeba D. (2021). A review on land use and land cover change in ethiopian basins. Land.

[33] Tsegaye K., Kendie H., Esa E. (2020). Soil erosion impact assessment using USLE/GIS approaches to identify high erosion risk areas in the lowland agricultural watershed of Blue Nile Basin , Ethiopia.

[34] Melese T., Senamaw A., Belay T., Bayable G. (2021). “The spatiotemporal dynamics of land use land cover change , and its impact on soil erosion in tagaw watershed. Blue Nile Basin , Ethiopia,”.

[35] Belay H.T., Malede D.A. (2020). Evaluating soil loss using geographical information system and remote sensing for soil and water resource conservation : the case of yisir watershed , northwestern Ethiopia.

[36] Assefa F., Elias E., Soromessa T., Aneseyee A.B. (2022).

[37] Ayele N.A., Naqvi H.R., Alemayehu D. (2022). Rainfall induced soil erosion assessment , prioritization and conservation treatment using RUSLE and SYI models in highland watershed of Ethiopia. Geocarto Int..

[38] Belayneh M., Yirgu T., Tsegaye D. (2019). Potential soil erosion estimation and area prioritization for better conservation planning in Gumara watershed using RUSLE and GIS techniques. Environ. Syst. Res..

[39] Gashaw T., Worqlul A.W., Dile Y.T., Addisu S., Bantider A., Zeleke G. (2020). Evaluating potential impacts of land management practices on soil erosion in the Gilgel Abay watershed , upper Blue Nile basin. Heliyon.

[40] Duguma T.A. (2022). Soil erosion risk assessment and treatment priority classi fi cation : a case study on guder watersheds , Abay river basin , Oromia , Ethiopia. Heliyon.

[41] Yeneneh N., Elias E., Feyisa G.L. (2022). Quantify soil erosion and sediment export in response to land use/cover change in the Suha watershed , northwestern highlands of Ethiopia : implications for watershed management. Environ. Syst. Res..

[42] Singh W.R., Barman S., Tirkey G. (2021). Morphometric analysis and watershed prioritization in relation to soil erosion in Dudhnai Watershed. Appl. Water Sci..

[43] Gela A.G., Mengistu D.A., Bekele D.A. (2023). Watershed prioritization for conservation planning using RUSLE and morphometric methods, Northwestern Ethiopia. J. Mt. Sci..

[44] Kushwaha N.L., Elbeltagi A., Mehan S., Malik A., Yousuf A. (2022). Comparative study on morphometric analysis and RUSLE-based approaches for micro-watershed prioritization using remote sensing and GIS. Arabian J. Geosci..

[45] Afework A., Minale A.S., Teketay D., Terefe B. (2023). Spatio-temporal dynamics of Acacia decurrens plantations in awi zone highlands , northwest Ethiopia. Pap. Appl. Geogr..

[46] Afework A., Minale A.S., Teketay D., Terefe B. (2023). Spatio-temporal dynamics of Acacia decurrens plantations in awi zone highlands, northwest Ethiopia. Pap. Appl. Geogr..

[47] Kimberlin L.W., Moldenhauer W.C. (1977).

[48] Kanito D., Bedadi B., Feyissa S. (2023). Sediment yield estimation in GIS environment using RUSLE and SDR model in Southern Ethiopia. Geomatics, Nat. Hazards Risk.

[49] Woldesenbet A.B., Wudmatas S.D., Denboba M.A. (2020). Enset - based land use land cover change detection and its impact on soil erosion in Meki river watershed , Western Lake Ziway Sub - basin , Central Rift Valley of Ethiopia. Environ. Syst. Res..

[50] Jothimani M., Getahun E., Abebe A. (2022). Remote sensing , GIS , and RUSLE in soil loss estimation in the Kulfo river.

[51] Olika G., Fikadu G., Gedefa B. (2023). GIS based soil loss assessment using RUSLE model : a case of Horo district , western Ethiopia. Heliyon.

[52] Borrelli P. (2021).

[53] Haregeweyn N. (2017). Comprehensive assessment of soil erosion risk for better land use planning in river basins : case study of the Upper Blue Nile River. Sci. Total Environ..

[54] Tesfaye G., Tibebe D., Agricultural J., Ababa A. (2019). Sediment yield source identification in gilgel gibe-I catchment using GIS-based RUSLE and SEDD models for soil conservation.

[55] Yadeta Saketa Kebede H.B.A., Tamene Endalamaw Nega, Sinshaw Berhanu G. (2021). Modeling soil erosion using RUSLE and GIS at watershed level in the upper. Environ. Challenges.

[56] Endalew T., Biru D. (2022). Soil erosion risk and sediment yield assessment with revised universal soil loss equation and GIS : the case of nesha watershed , southwestern Ethiopia. Results Geophys. Sci..

[57] Terefe T.B., Baye Tadele Melese, Tsegaye Aderaw, Amene Afework, Yibeltal Tihtinaw, Anagaw Abebe, Temesgen Fekadu (2023). Review of soil loss estimation in Ethiopia : evaluating the use of the RUSLE model integrated with GIS and remote sensing techniques. Res. Sq..

[58] Bayable G. (2023). Detection of water hyacinth (Eichhornia crassipes) in lake tana, Ethiopia, using machine learning algorithms. Water (Switzerland).

[59] Molla T., Sisheber B. (2017).

[60] Kidane M., Bezie A., Kesete N., Tolessa T. (2019). The impact of land use and land cover (LULC) dynamics on soil erosion and sediment yield in Ethiopia. Heliyon.

[61] Yebyo S.G. (2022).

[62] Atoma H., V Suryabhagavan K., Balakrishnan M. (January, 2020). Soil erosion assessment using RUSLE model and GIS in Huluka watershed , Central Ethiopia. Sustain. Water Resour. Manag..

[63] Tessema I., Simane B., Angassa K. (2023). Soil erosion estimation and risk assessment at watershed level : a case study of Neshe Dam Watershed in Blue Nile River basin , Ethiopia. Int. J. River Basin Manag..

[64] Singh W.R., Barman S., Patidar N. (2021). “Prioritization of sub-watersheds based on morphometric parameters in pare watershed. Arunachal Pradesh , India,”.

[65] Bharath A., Kumar K.K., Maddamsetty R., Manjunatha M., Tangadagi R.B. (2021). Drainage morphometry based sub-watershed prioritization of Kalinadi basin using geospatial technology. Environ. Challenges.

[66] Rahmati O. (2019). Swpt : an automated GIS-based tool for prioritization of sub-watersheds based on morphometric and topo-hydrological factors.

[67] Römping U.G. (2009).

[68] Lovatti B.P.O., Nascimento M.H.C., Neto Á.C., Castro E.V.R., Filgueiras P.R. (2019). Use of Random forest in the identification of important variables. Microchem. J..

[69] Bland J.M., Altman D.G. (Feb. 1986). Statistical methods for assessing agreement between two methods of clinical measurement. Lancet (London, England).

[70] Esa E., Assen M., Legass A. (2018). Implications of land use/cover dynamics on soil erosion potential of agricultural watershed , northwestern highlands of Ethiopia. Environ. Syst. Res..

[71] Tsegaye L., Bharti R. (2021). Soil erosion and sediment yield assessment using RUSLE and GIS - based approach in Anjeb watershed , Northwest Ethiopia. SN Appl. Sci..

[72] Degife A., Worku H., Gizaw S. (2021). Environmental implications of soil erosion and sediment yield in Lake Hawassa watershed , south - central Ethiopia. Environ. Syst. Res..

[73] Belay T., Mengistu D.A. (2021). Impacts of land use/land cover and climate changes on soil erosion in Muga watershed , Upper Blue Nile basin (Abay), Ethiopia. Ecol. Process..

[74] Eniyew S., Teshome M., Sisay E., Bezabih T. (2021). Integrating RUSLE model with remote sensing and GIS for evaluation soil erosion in Telkwonz Watershed , Northwestern Ethiopia. Remote Sens. Appl. Soc. Environ..

[75] H, Blanco R.L. (2021).

[76] Stocking M. (2002).

[77] Boardman J. (2023).

[78] Nguyen X.H. (2018).

[79] Sharma N., Yousuf A., Kaushal A. (2023).

[80] Meshram U. (2021). “Watershed prioritization using morphometric parameters in some part of Painganga River sub- basin , Maharashtra , India Watershed prioritization using morphometric parameters in some part of Painganga River sub- basin. Maharashtra , India,”.

[81] Negese A. (2021). Impacts of land use and land cover change on soil erosion and hydrological responses in Ethiopia. Appl. Environ. Soil Sci..

[82] Ayalew G. (2015). A Geographic information system based soil loss and sediment estimation in Zingin watershed for conservation planning , highlands of Ethiopia.

[83] Desalegn M.Y., Miheretu B.A., Gobezie T. (2023). Impact of land use/land cover changes on soil erosion risk in upper Mile River sub-watershed , North Eastern highlands of Ethiopia. Geol. Ecol. Landscapes.

